# Standardization and harmonization in clinical dosimetry in terms of absorbed dose to water as the measurand for beta radiation brachytherapy

**DOI:** 10.4103/0971-6203.26688

**Published:** 2006

**Authors:** U Quast

**Affiliations:** Editorial Board Member, Journal of Medical Physics, Formerly, Department of Radiotherapy, Essen University Hospital, Essen, Germany

Beta radiation has been applied successfully since a long time for brachytherapy treatment of small- and medium-sized intraocular tumors, especially for malignant melanoma of the chorioidea utilizing ^106^Ru/^106^Rh sources. Recently (since the mid-1990s), beta radiation sources have found promising applications in intravascular brachytherapy for the prevention of restenosis (recurrent stenosis) of coronary arteries after angioplasty. Numerous new beta radiation sources have been developed. This rapidly increasing use, worldwide, demands international standardization and harmonization in clinical beta radiation dosimetry.[1] While IAEA-TECDOC-1274 (2002)[2] still recommends well-type ionization chambers as working standards for calibration, there is the need and possibility to calibrate all beta radiation brachytherapy sources in terms of absorbed dose to water at the clinically relevant reference distance of 2 mm in water for ophthalmic applicators and intracoronary brachytherapy sources (and at 5 mm for sources for brachytherapy in peripheral vessels). New methods of adequate clinical dosimetry and some degree of skill and knowledge are required for the application of beta radiation sources for brachytherapy as very strong fluence gradients are present in the immediate vicinity of the source. (At 2 mm distance in water, the dose typically changes by a factor of 2 within 1 mm.) Efforts have to be accelerated to develop primary, secondary and transfer standards as well as appropriate dosemeters to ensure the required accuracy.

Beta dosimetry has seen a significant progress as well as methods of dose calculation as it poses challenge to both. Traditional methods, the integration of beta radiation point-source dose functions or point dose kernels, have been widely applied but cannot generally provide results of sufficient accuracy. For the calculations of dose distributions, Monte Carlo simulation methods have been shown to exhibit promising tools. The need for accurate experimental dose measurements, however, cannot be compromised - not only for the audit and validation of the calculations but also for routine dosimetry. The newly developed 40 channel multielectrode extrapolation chamber equipped with a matrix of 1 mm^2^ square collecting electrodes is now in use as primary standard at the PTB to calibrate beta radiation sources in terms of absorbed dose to water at the reference depth of 2 mm in water-equivalent material as well to measure full 3D absorbed dose distributions within a short time. For routine dosimetry, radiochromic dye films are used to characterize beta radiation source uniformity and micro-plastic scintillator detector systems (including a NIST-calibrated ^90^Sr source and an automated 3D water phantom) allow precise, fast 3D absorbed dose measurements. Further, various detectors, including small-volume ionization chambers and tiny TLDs, continue making great news.

The measurand absorbed dose to water is now in use for external beam radiotherapy worldwide. This clinically relevant dose quantity must also be employed as the measurand for radiotherapy in general, thus including brachytherapy. Supported by the development of new primary and secondary standards for realizing and disseminating the Gray for the measurand absorbed dose to water for all types and qualities of therapeutic radiation, the next step has to be its introduction to brachytherapy with beta radiation. In this direction, it is relevant to mention that the German standardization organization in radiology, DIN NAR task group dosimetry, established an international ad hoc working group in 2002 to prepare a New Work Item Proposal for an ISO Standard on *Clinical dosimetry – Beta radiation sources for brachytherapy*, based on the report of the AAPM radiation therapy task group No. 60 (1999),[3] on the German DGMP report No. 16 (2001),[4] on the ICRU report No. 72 (2004)[5] and other documents. Based on the recommendations of this working group, later in 2004, an ISO Project led by NIST, USA, was launched within ISO TC85 / SC2 / WG22 *(Medical Physics)* / SG2. In 2006, the committee draft for vote was accepted.[6] The F-DIS is planned for the end of this year and the standard should be ready in 2007/2008.

This new ISO standard would specify the methods for the determination of the measurand absorbed dose to water for beta radiation sources. Topics to be covered in this ISO standard are terms and definitions, beta radiation sources, dose calculation parameters and formalisms, calibration and traceability, dose measurements in phantoms and measurement corrections, theoretical modeling, uncertainties in source dosimetry calibrations, treatment planning and reporting and clinical quality control. Detailed guidance is also planned to be given by the annex covering the topics source reference data, primary standards for beta radiation dosimetry, detectors and phantom materials and Monte Carlo calculations. The standard would be confined to ‘sealed’ beta radiation sources, such as plenary and concave surface sources, single seeds and source trains, line sources and shell and volume sources for which the beta radiation emitted is of therapeutic relevance.

It is hoped that the final step of introducing the absorbed dose to water as the measurand in radiotherapy in general could be achieved by a further new ISO project on: *Clinical dosimetry – Medium and low energy photon radiation sources for brachytherapy*. Let us look forward to another milestone to be cemented on the road of achieving higher precision and accuracy in this field of science devoted to health care of the humanity.
